# Nutritional and Therapeutic Potential of *Stropharia rugosoannulata* and *Macrolepiota procera*: From Composition to Health-Promoting Effect

**DOI:** 10.3390/jof11040259

**Published:** 2025-03-27

**Authors:** Qian Wang, Xiaoyan Yang, Jiangxiong Zhu

**Affiliations:** 1 College of Biomedicine and Health, Anhui Science and Technology University, Fengyang 233100, China; qianwang420@163.com; 2 College of Agriculture, Anhui Science and Technology University, Fengyang 233100, China; 3 School of Agriculture and Biology, Shanghai Jiao Tong University, Shanghai 200240, China

**Keywords:** *Stropharia rugosoannulata*, *Macrolepiota procera*, bioactivity, extraction methods, safety, toxicity

## Abstract

*Stropharia rugosoannulata* and *Macrolepiota procera* have garnered considerable attention due to their distinctive flavor profile, culinary versatility, and potential nutritional and therapeutic benefits. They are a rich source of high-quality protein, dietary fiber, vitamins, and minerals, contributing to daily nutritional requirements and promoting overall well-being. Furthermore, they contain a diverse array of bioactive compounds, including polyphenols, flavonoids, and triterpenoids, which have demonstrated antioxidant, anti-inflammatory, and antitumor properties in previous studies. However, comprehensive reviews focusing on these two species remain limited. Therefore, this review summarizes the types of nutrients and bioactive compounds found in *Stropharia rugosoannulata* and *Macrolepiota procera*, along with their respective extraction methods. Moreover, the bioactivities of these compounds were discussed, aiming to provide a theoretical framework for the development of novel functional foods and nutraceuticals derived from *Stropharia rugosoannulata* and *Macrolepiota procera*.

## 1. Introduction

**Figure 1:**
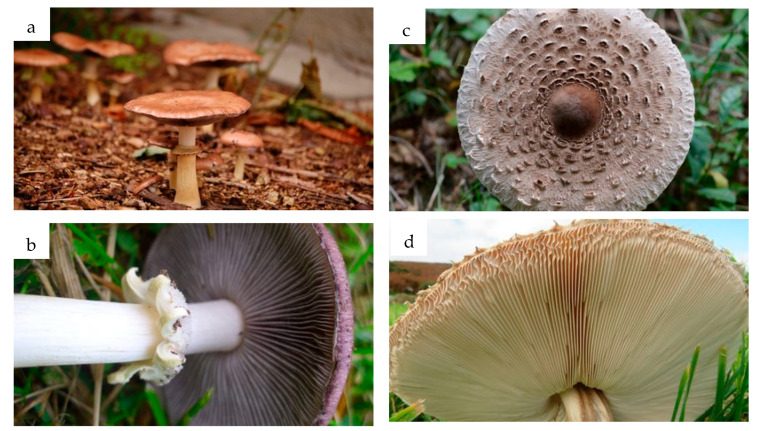
Morphology of *S. rugosoannulata* (**a**,**b**) and *M. procera* (**c**,**d**). (https://ultimate-mushroom.com/edible/224-stropharia-rugosoannulata.html (accessed on 26 November 2024); https://ultimate-mushroom.com/edible/209-macrolepiota-procera.html (accessed on 26 November 2024); Permission to reproduce these images has been obtained).

Edible mushrooms are prized for their delicate flavor and rich nutritional profile, including high-quality protein, vitamins, minerals, and dietary fiber, making them a promising candidate as a future health food [1]. Two prominent species of this group are the *Stropharia rugosoannulata* Farl. ex Murrill (*S. rugosoannulata*) and the *Macrolepiota procera* (Scop.) Singer (*M. procera*), which both belong to the *Agaricales* order, has garnered significant scientific interest due to their low-calorie, high-protein, low-fat, low-cholesterol, and vitamin-rich composition, highlighting their potential nutritional and medicinal properties [2]. Figure 1 depicts the morphology of these two species.

*S. rugosoannulata*, a medium-sized mushroom, is readily identifiable in the wild by the wrinkled annular zone on its cap. Cap color ranges from pale yellow to brown, with white to cream-colored gills. The stipe is long and slender, reaching 9–15 cm at maturity, and its surface is smooth or finely scaled [3]. *S. rugosoannulata* is distributed across temperate and subtropical regions, including China, Japan, Korea, Germany, the United Kingdom, France, and Italy, as well as parts of the United States, Canada, South America, and Australia [4]. Within China, it is commonly found in the deciduous broadleaf and mixed coniferous-deciduous forests of the northeast, north, and southwest regions [5]. As a saprophytic fungus, *S. rugosoannulata* plays a crucial role in forest ecosystems by decomposing leaf litter and efficiently degrading various organic wastes, thereby accelerating nutrient cycling and enhancing soil fertility [6,7,8,9]. Furthermore, it can form symbiotic relationships with certain trees, facilitating water and mineral uptake by the host plant and improving its stress resistance and growth.

First cultivated in Germany in 1969, *S. rugosoannulata* was subsequently introduced to other European countries [10]. China introduced the species from Poland in 1980, with successful cultivation trials in Fujian, Zhejiang, and Guangxi provinces, leading to its rapid expansion and widespread cultivation nationwide [11,12]. Established cultivation techniques utilize various methods, including open fields, forests, pots, and greenhouses, employing substrates such as starch (carbon source), urea (nitrogen source), and corn stalks. Optimal growth conditions include temperatures between 20 and 30 °C and relative humidity of 70–85% [13], along with appropriate shade, adequate ventilation, and sufficient mineral nutrients. Yield and quality are influenced by factors such as substrate composition, temperature, humidity, and light. Due to its simple cultivation process, diverse cultivation models, low-cost, and feasibility, *S. rugosoannulata* holds significant promise for commercial production.

*M. procera*, a large edible mushroom, has an off-white fruiting body with a stipe characteristically adorned with brown scales arranged in a snakeskin pattern. The prominent annulus is mobile. Gills are white and free. The cap surface features patchy scales composed of slightly thick-walled, hair-like, yellowish-brown hyphae. The flesh is thick and flavorful [14]. *M. procera* is primarily found in grasslands, forest edges, and open areas of Germany, France, Italy, and the United Kingdom; moist grasslands and forest margins of the United States and Canada; and parts of China, Japan, and Korea [15]. In China, it is distributed in the mountainous regions of East, Central, and Southwest China, frequently appearing in forests after summer rains. As an ectomycorrhizal fungus, *M. procera* forms symbiotic associations with various tree species, enhancing nutrient acquisition and disease resistance in host plants, and contributing to the health and stability of forest ecosystems [16]. Furthermore, *M. procera* is a high-quality edible mushroom, appreciated for its tender texture, delicious taste, rich nutritional content, and high levels of essential amino acids, making it a promising candidate for further development and utilization, with potential positive impacts on local economies and cultural practices [17].

In some regions, *M. procera* is not yet commercially cultivated and is collected from the wild during the harvest season. Cultivation research is largely concentrated in Turkey, involving processes such as composting, pre-composting fermentation, spawning, and casing. Peksen et al. [18] identified wheat as the optimal grain for *M. procera* spawn production, with mycelial growth observed on substrates prepared from wheat straw, peat, oak leaves, mixtures of wheat straw and peat, oak leaves and peat, and oak leaves and wheat bran; however, fruiting was not achieved. Peksen and Kibar [19] reported optimal mycelial growth at pH 6.0, 25 °C, with glucose as the carbon source and yeast extract and peptone as nitrogen sources. Adamska et al. [14] noted that *M. procera*, typically found in the wild, exhibits optimal laboratory growth conditions at 30 °C on potato dextrose agar (PDA) at pH 7.0, utilizing maltose as the carbon source, glycine as the nitrogen source, a C:N ratio of 10:1, and a medium supplemented with 1% glucose.

Both *S. rugosoannulata* and *M. procera* are not only ecologically significant but also possess substantial nutritional and medicinal potential. *S. rugosoannulata* is rich in protein, containing a complete profile of essential amino acids, and is a good source of beta-glucans and other polysaccharides, B vitamins, vitamin D, and minerals such as calcium, iron, and magnesium [20]. It also contains various bioactive compounds, including triterpenoids and phenolic compounds [21,22,23], contributing to its notable nutritional value. These components confer immunomodulatory, antitumor, and antioxidant properties, suggesting potential applications in disease prevention and treatment. Similarly, *M. procera* boasts high protein content, a complete array of essential amino acids, digestive-aid dietary fiber, vitamins B2, B3, and B5, minerals such as potassium, phosphorus, magnesium, and iron, as well as bioactive compounds like polyphenols and flavonoids [24], all contributing to its high nutritional value. These constituents suggest potential immunomodulatory, antitumor, antioxidant, and lipid-lowering properties. Both species are not only culinary delicacies but also valuable natural medicinal resources, offering diverse health benefits.

Given the growing interest in the medicinal and nutritional properties of *S. rugosoannulata* and *M. procera*, this review summarizes the current research on them, focusing on their nutritional composition, bioactive compounds, extraction methods, safety, and toxicity, aiming to provide a robust scientific foundation for understanding and utilizing the nutritional and medicinal value of these edible fungi.

## 2. Nutritional Composition

**Figure 2:**
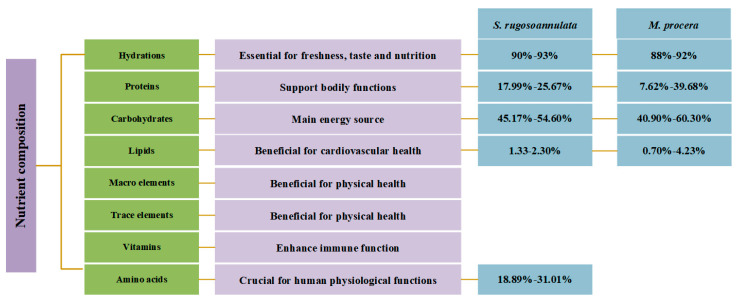
Nutrient composition of *S. rugosoannulata* and *M. procera* (dw%). Nutrients without listed percentages indicate either minimal content or a lack of specific data.

The variety of nutrients makes the edible mushrooms produce a variety of flavors and textures [25]. While *S. rugosoannulata* and *M. procera* share many similarities, they also exhibit differences in moisture, protein, carbohydrate (including dietary fiber), and lipid (fatty acid profile) content. Understanding these variations is crucial for optimizing dietary choices and maximizing the nutritional benefits derived from consuming these mushrooms are fundamental to life and must be obtained through dietary intake. *S. rugosoannulata* and *M. procera*, recognized for both their edibility and medicinal properties, are rich sources of various nutrients, including proteins, carbohydrates, lipids, minerals, vitamins, and amino acids (Figure 2, Table 1). Additionally, components such as nucleotides, free amino acids, soluble sugars, and organic acids contribute to various flavors and textures [25].

### 2.1. Moisture

Moisture is the major component of mushrooms and significantly influences their freshness, texture, and nutritional value. *S. rugosoannulata* typically exhibits a moisture content of 90–93%, contributing to its tender texture when fresh but also rendering it susceptible to spoilage during prolonged storage [3]. *M. procera* shows a similar moisture profile, generally ranging from 88 to 92%. Due to its larger fruiting body and consequently faster surface evaporation, careful humidity control is essential during storage.

### 2.2. Protein and Amino Acids

Proteins are crucial components of mushrooms and provide essential amino acids and support various bodily functions. *S. rugosoannulata* and *M. procera*, being popular edible mushrooms, have attracted considerable attention in food and nutritional science. Amino acids, the building blocks of proteins, are vital for normal physiological function. Essential amino acids cannot be synthesized by the human body and must be obtained through dietary sources, whereas non-essential amino acids can be synthesized endogenously.

*S. rugosoannulata* is characterized by a relatively high protein content, comprising a diverse range of proteins and a complete profile of essential amino acids [26]. Recent studies report a total amino acid content of 18.89–31.01% in *S. rugosoannulata*, with 18 of the 20 proteinogenic amino acids present, including 6.43–11.70% essential amino acids [26,44,45]. Isoleucine, the most abundant essential amino acid, plays a crucial role in protein synthesis and muscle protein metabolism [46]. *S. rugosoannulata* is also rich in non-essential amino acids, particularly glutamic acid and aspartic acid. Glutamic acid, typically present at 2.88–6.84% [27], functions as an important neurotransmitter and participates in energy metabolism and immune regulation. Aspartic acid, also present in relatively high amounts (1.72–3.07%), contributes to energy production and muscle repair [45].

While *M. procera* contains a slightly higher protein content than *S. rugosoannulata*, it still offers a diverse amino acid profile, albeit with potentially lower bioavailability. Their essential amino acid content is relatively lower overall but notable for specific amino acids. Although lysine levels in *M. procera* are lower than in *S. rugosoannulata*, its overall essential amino acid composition still provides valuable nutritional support. *M. procera* is also rich in non-essential amino acids, notably alanine and tyrosine. Studies indicate an alanine content of 1.10 g/100 g in *M. procera* [47], contributing to muscle metabolism and immune function. Tyrosine, essential for nervous system health, serves as a precursor for various neurotransmitters.

### 2.3. Carbohydrates

Carbohydrates, the primary energy source for mushrooms, are mainly composed of soluble and insoluble fiber and other polysaccharides. *S. rugosoannulata* exhibits a relatively high carbohydrate content, accounting for 45.17–54.60% of its dry weight, with a substantial portion attributed to dietary fiber (5.25–7.99%), particularly health-promoting polysaccharides like β-glucans [29]. The dietary fiber content of 2–3% is beneficial for digestive health. In contrast, *M. procera* has a higher carbohydrate content (40.90–60.30% dry weight) [30], with a correspondingly lower dietary fiber content, although it still plays a significant role in modulating gut microbiota and promoting intestinal health [28].

### 2.4. Lipids

While lipids constitute a minor component of mushrooms, their composition has important implications for health. *S. rugosoannulata* typically contains 1.33–2.30% lipids, with unsaturated fatty acids comprising over 77% of the total fatty acid content, including omega-6 and smaller amounts of omega-3 fatty acids [29]. Linoleic acid (C18:2), a polyunsaturated omega-6 fatty acid, is the most abundant, representing over 57% of total fatty acids. Among saturated fatty acids, palmitic acid (C16:0) predominates, accounting for over 13% [48]. Other fatty acids present include oleic acid (C18:1), palmitoleic acid (C16:1), stearic acid (C18:0), and lignoceric acid (C24:0). This fatty acid profile is considered beneficial for cardiovascular health.

*M. procera* contains approximately 0.70–4.23% lipids, with a fatty acid profile predominantly composed of polyunsaturated fatty acids [31], which play important roles in anti-inflammatory and immunomodulatory processes. Saturated fatty acids account for 15.9%, while unsaturated fatty acids represent 81.9% of the total. Linoleic acid (62.4%) is the dominant unsaturated fatty acid, followed by smaller proportions of oleic acid (17.4%) and palmitic acid (10.9%) [49].

### 2.5. Minerals

#### 2.5.1. Macroelements

Potassium, phosphorus, and magnesium are the primary macronutrients in *S. rugosoannulata*. Potassium is particularly abundant, at approximately 1600 mg/100 g, contributing to heart health, blood pressure regulation, and fluid balance [32]. Phosphorus (75–100 mg/100 g) supports bone and teeth health, while magnesium (20–30 mg/100 g) is essential for bone health and muscle function. Calcium content is around 70–80 mg/100 g [33]. *M. procera* is also rich in macronutrients, with a substantial potassium content (300–500 mg/100 g), serving as a good dietary source of this mineral [50]. Magnesium levels are lower (15–25 mg/100 g), while calcium content is within the typical range (400–500 mg/100 g) [34].

#### 2.5.2. Trace Elements

Iron is a notable micronutrient in *S. rugosoannulata*, with levels of 19.5–24.5 mg/100 g, contributing to daily iron requirements [35]. Zinc (5.5–10.0 mg/100 g) supports immune function and cell regeneration [36], while selenium (0.1–0.5 mg/100 g) acts as an antioxidant, promoting cellular health [37,38]. *M. procera* contains slightly higher levels of iron (1.5–3 mg/100 g) compared to *S. rugosoannulata*. Zinc content is similar (0.5–1.2 mg/100 g). Copper, important for antioxidant defense, is present in relatively high amounts in *M. procera* [39].

### 2.6. Vitamins

*S. rugosoannulata* and *M. procera* exhibit distinct vitamin profiles. *S. rugosoannulata* contains relatively low levels of various water-soluble B vitamins, including riboflavin (B2), niacin (B3), pantothenic acid (B5), pyridoxine (B6), folate (B9), and vitamin B12. Studies suggest that the polysaccharides and antioxidants present in this species may enhance B vitamin absorption [20]. It also contains vitamin A [51], α-tocopherol (vitamin E) [40], and vitamin C (ascorbic acid) [41], as well as fat-soluble vitamins such as ergosterol, a precursor to vitamin D2, and relatively low levels of carotenoids (vitamin A precursors). The low carotenoid content limits its potential for vitamin A conversion.

*M. procera* is a good source of B vitamins, especially riboflavin (B2), niacin (B3), and pantothenic acid (B5), which play important roles in energy metabolism, skin health, and nervous system function [42]. It typically contains higher levels of vitamin D, particularly after exposure to sunlight, and the presence of its high content of ergosterol significantly enhances vitamin D2 (ergocalciferol) synthesis [52]. *M. procera* also contains vitamin E, contributing to antioxidant defense and cardiovascular health; vitamin K, essential for blood clotting; and carotenoids. Its deeper color suggests a potentially higher content of plant pigments.

## 3. Bioactive Compounds

### 3.1. Polysaccharides

**Figure 3:**
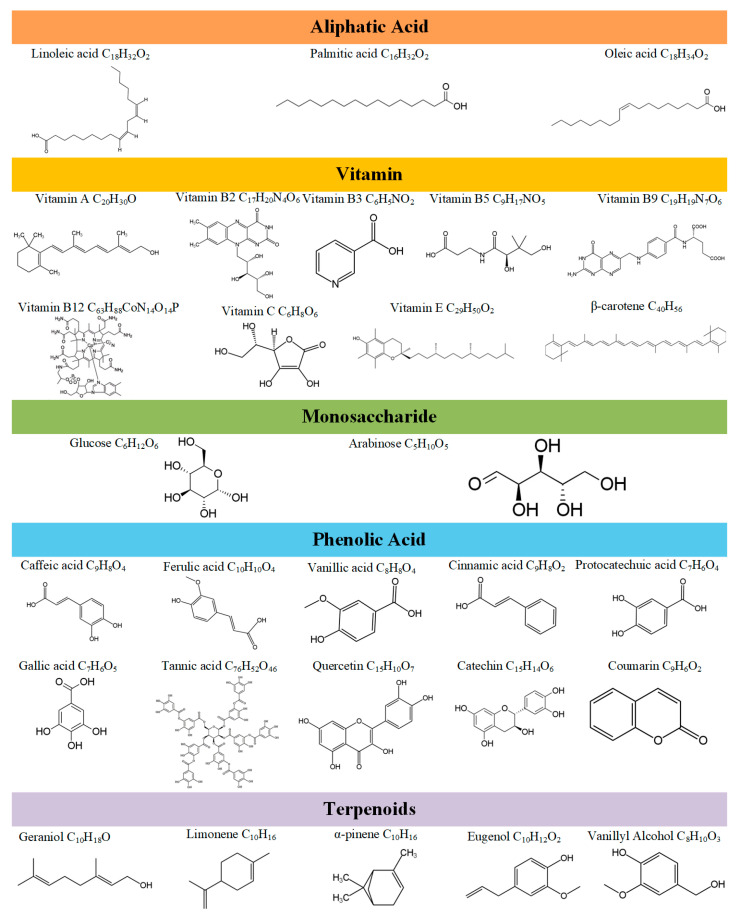
Bioactive compound profiles of *S. rugosoannulata* and *M. procera*.

Polysaccharides, complex carbohydrates composed of multiple monosaccharides linked by glycosidic bonds, are ubiquitous in fungi. These macromolecules are recognized for their significant health benefits, including antioxidant, antidiabetic, immunomodulatory, antitumor, anti-inflammatory, and hypoglycemic activities [53]. *S. rugosoannulata* and *M. procera* are rich sources of polysaccharides, contributing to their nutritional and medicinal value. Figure 3 illustrates the composition of some bioactive compounds in *S. rugosoannulata* and *M. procera*.

**Figure 4:**
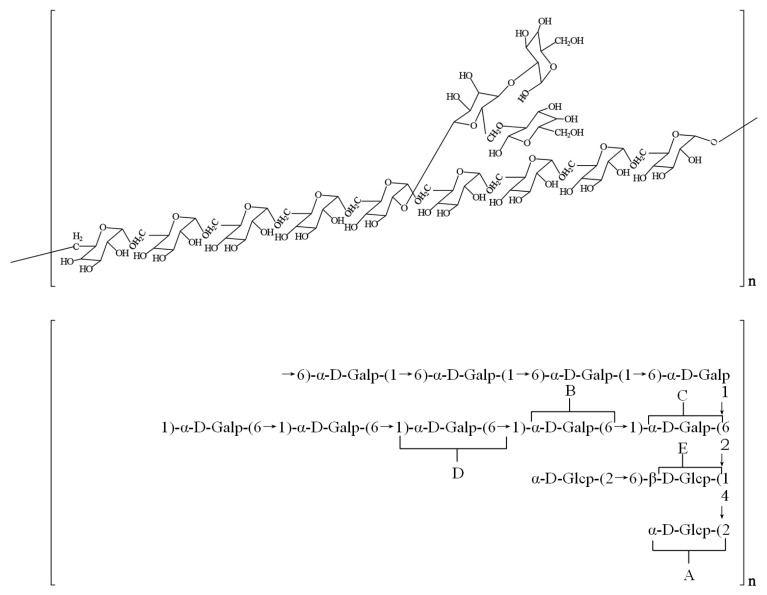
Predicted structure of *S. rugosoannulata* polysaccharide SR-1 [59]. A: α-D-Glcp(2→(A); B: 6→)-α-D-Galp(1→(B); C: 2, 6→)-α-D-Galp(1→(C); D: 6→)-α-D-Galp(1→(D); E: 4, 6→)-β-D-Glcp(1→(E).

Soluble polysaccharides, primarily heteropolysaccharides, are the most prevalent type in *S. rugosoannulata* [54]. Jiang et al. [55] isolated a novel polysaccharide, SRF-3, from *S. rugosoannulata*. SRF-3, with an average molecular weight of approximately 24 kDa, is composed of mannose, galactose, methyl galactose, and fructose in a ratio of 16:12:58:12. Its main chain consists of repeating α-D-1,6-Galp and α-D-1,6-Me-Galp units, with branching at the O-2 position of galactose. This structure is proposed to be a mannogalactan with minor t-β-D-Manp side chains. Monosaccharides detected in *S. rugosoannulata* include glucose, galactose, and mannose, with glucose being the most abundant [56]. Based on the presence or absence of glucuronic acid or sulfate groups, *S. rugosoannulata* polysaccharides can be classified as neutral or acidic [56,57,58]. Neutral polysaccharides primarily consist of (1→6)-α-d-glucan or (1→6)-α-d-galactan backbones, while acidic polysaccharides feature (1→6)-α-d-glucan or (1→3)-β-d-glucan backbones [56,58]. Both types contain α and β glycosidic linkages. Jiang [59] identified a novel polysaccharide, SR-1, in *S. rugosoannulata*, composed of galactose and glucose in a 3:1 ratio, with a (1→6)-α-D-galactose and (1→6, 2)-α-D-galactose backbone and side chains of (1→6, 4)-β-D-glucose attached to the 2-O position. The glucose residues in the side chains are further linked to →2)-α-D-glucopyranose at the 4-O and 6-O positions (Figure 4).

*M. procera* also contains β-glucans, characterized by relatively low molecular weight and high branching, primarily through β-1,3 and β-1,4 glycosidic linkages, resulting in a complex three-dimensional structure [60]. This branching pattern contributes to its biocompatibility and facilitates effective binding to immune cells, enhancing immune responses. Polysaccharides in *M. procera* are composed of various sugar units, including glucose, mannose, arabinose, and galactose, arranged in complex glycan structures with varying proportions. These polysaccharides typically feature β-(1→3)-linked glucose chains with diverse side chains, potentially influencing their biological activity. *M. procera* predominantly contains neutral polysaccharides, with glucose (Glc) being the major monosaccharide component (62.3% *w*/*w*). Other linear and/or branched glucan-type polysaccharides may also be present. In addition to Glc, the polysaccharides are rich in galactose (Gal, 19.7% *w*/*w*) and contain the less common 3-O-Me-Gal (2.7% *w*/*w*). The presence of mannose (Man, 6.9% *w*/*w*) and fucose (Fuc, 3.4% *w*/*w*) suggests the potential formation of heteropolysaccharides. Galacturonic acid (GalA) and glucuronic acid (GlcA) have also been detected. *M. procera* accumulates substantial amounts of water-soluble α-glucans and/or glycogen, and its (hetero) β-glucans contain 1,4,6-β-D-Manp structures [61].

### 3.2. Phenolic Compounds

Phenolic compounds, a diverse class of natural products widely distributed in plants, exhibit a wide range of bioactivities, including antioxidant, anti-inflammatory, antitumor, and antimicrobial effects. *S. rugosoannulata* and *M. procera* are rich in phenolic compounds, which contribute to their unique flavor and color profiles and offer significant health benefits, particularly in terms of antioxidant and anti-inflammatory properties.

Major phenolic acids in *S. rugosoannulata* include caffeic acid, ferulic acid, and vanillic acid. The phenolic acid content is influenced by environmental factors, harvest time, and processing methods. Caffeic acid levels can reach 1.5 mg/g, while ferulic acid is present at approximately 0.8 mg/g. These phenolic acids exhibit strong antioxidant activity, effectively scavenging free radicals and mitigating oxidative damage [62]. Flavonoids in *S. rugosoannulata* include quercetin, catechin, and flavonols. Quercetin content can reach 0.5 mg/g, while catechin levels are around 0.3 mg/g. These flavonoids possess antioxidant, anti-inflammatory, and antitumor activities [63]. In addition to phenolic acids and flavonoids, *S. rugosoannulata* contains other phenolic derivatives, such as coumarins and tannins, which exhibit antioxidant and antimicrobial properties, with tannins also demonstrating inhibitory effects on certain cancer cells.

*M. procera* is also rich in phenolic acids. Analysis has revealed vanillic acid as the major component, followed by cinnamic acid, protocatechuic acid, and gallic acid. The antioxidant capacity of these phenolic acids suggests potential applications in food preservation and nutritional supplementation [64]. *M. procera* contains high concentrations of polyphenols, which contribute significantly to its antioxidant capacity, effectively scavenging free radicals and potentially reducing the risk of cardiovascular diseases and certain cancers [65]. Flavonoids in *M. procera* include quercetin, flavonols, and isorhamnetin. Quercetin content can reach 0.7 mg/g, while flavonol levels are around 0.4 mg/g. The bioactivity of these flavonoids has made *M. procera* a focus of nutritional and pharmacological research [66]. *M. procera* also contains coumarins and tannins. Coumarins, present in relatively high amounts, exhibit antioxidant and antimicrobial properties, while tannins show potential for inhibiting tumor cell growth.

### 3.3. Terpenes

Terpenes, a diverse class of natural products widely found in fungi, are renowned for their different structures and significant biological activities, with applications in medicine, perfumery, and the food industry. In *S. rugosoannulata* and *M. procera*, terpenes occur as specialized secondary metabolites, encompassing monoterpenes, sesquiterpenes, triterpenes, and steroids, contributing to their medicinal potential.

Major monoterpenes in *S. rugosoannulata* include geraniol and limonene. Geraniol exhibits antibacterial and antioxidant properties, effectively inhibiting the growth of various pathogens. Limonene possesses anti-inflammatory and analgesic effects, potentially benefiting digestive function. Sesquiterpenes in *S. rugosoannulata* primarily include β-caryophyllene and α-pinene. β-Caryophyllene, with its distinctive aroma and bioactivity, is widely used in the fragrance industry [67]. α-Pinene exhibits antioxidant and antitumor activities, promoting cellular health [68]. While triterpenes are relatively less abundant in *S. rugosoannulata*, bioactive triterpenes such as β-sitosterol have been identified. β-Sitosterol is recognized for its anti-inflammatory, lipid-lowering, and anticancer properties [69]. Steroids, such as sterols, which regulate cell membrane fluidity and stability, also exhibit antioxidant and anti-inflammatory activities [66].

Monoterpenes in *M. procera* include geraniol and eugenol. Geraniol, present in higher concentrations, exhibits anticancer and antioxidant properties [70]. Eugenol, known for its strong aroma and antimicrobial activity, is widely used in food and pharmaceuticals [71]. Sesquiterpenes in *M. procera* primarily include α-pinene and β-caryophyllene. α-Pinene has significant antioxidant and anti-inflammatory effects [68], while β-caryophyllene demonstrates anticancer activity, inhibiting the proliferation of certain cancer cells [67]. *M. procera* is also rich in triterpenes, such as β-sitosterol and lanosterol. β-Sitosterol, present in substantial amounts, exhibits lipid-lowering and anti-inflammatory effects [72]. Lanosterol is recognized for its antioxidant and antitumor activities [73]. Sterols are the primary steroids in *M. procera*, potentially contributing to cardiovascular health and cholesterol regulation.

### 3.4. Polypeptides and Enzymes

Research on polypeptides and enzymes in *S. rugosoannulata* and *M. procera* has intensified in recent years, highlighting their roles in various physiological processes and their potential health benefits, with promising applications in functional foods and natural medicines.

Bioactive peptides identified in *S. rugosoannulata* often exhibit antioxidant, antimicrobial, and immunomodulatory functions. Studies have shown that *S. rugosoannulata* is rich in specific small peptides with significant antioxidant capacity. Bioactive oligopeptides from *S. rugosoannulata* fruiting bodies possess antioxidant and angiotensin-converting enzyme (ACE) inhibitory activities. Optimal extraction conditions involve pure water, a 1:20 (*w*/*v*) ratio, and ultrasonication at 120–400 W and 20 kHz for 10–35 min, resulting in a yield of 11.04–23.02% [74]. Research on flavor peptides from *S. rugosoannulata*, focusing on preparation techniques, structure–activity relationships, and flavor perception mechanisms, has revealed that free peptides can constitute 11–14% of the dry weight of mature fruiting bodies, significantly higher than free amino acids and nucleotides. Over 50% of these peptides exhibit salty and umami characteristics. Glu148, Glu301, Glu48, and Ala46 are key active amino acid residues involved in binding to T1R1/T1R3 taste receptors [23,74,75]. *S. rugosoannulata* also contains various enzymes that facilitate nutrient digestion and absorption and play crucial roles in antioxidant processes. Peroxidases, for example, scavenge free radicals, protecting cells from oxidative damage, with potential applications in food preservation and medicine [76].

*M. procera* is also rich in bioavailable peptides. Some of these peptides exhibit strong antioxidant and antimicrobial activities, effectively scavenging free radicals and inhibiting the growth of various pathogens. Its enzyme system includes proteases, lipases, and amylases, which are essential for growth and development and facilitate nutrient digestion and absorption. Proteases from *M. procera* can degrade antinutritional factors in food, improving protein bioavailability. Lipases play a crucial role in lipid metabolism, breaking down complex fats, with potential benefits for cardiovascular health [77].

### 3.5. Other Bioactive Compounds

In addition to the compounds mentioned above, *S. rugosoannulata* contains various alcohols with antioxidant and anti-inflammatory activities, potentially reducing the risk of chronic diseases. Its mycelium contains 0.016–0.020% alkaloids and 0.35–0.41% flavonoids [78]. *M. procera* also contains specialized secondary metabolites, such as alkaloids and flavonoids, which exhibit antibacterial, antiviral, and antitumor activities, suggesting potential applications in viral disease prevention.

## 4. Preparation of Bioactive Compounds

Efficient extraction techniques are crucial for maximizing the yield and purity of bioactive compounds from *S. rugosoannulata* and *M. procera*. While traditional methods like solvent and hot water extraction have long been dominant, modern techniques such as ultrasound-assisted extraction (UAE) and microwave-assisted extraction (MAE) are gaining prominence. Furthermore, when the purity of extracted compounds is insufficient, researchers often employ analytical techniques like high-performance liquid chromatography (HPLC), gas chromatography (GC), and preparative chromatography for purification and characterization. Table 2 summarizes the extraction and isolation methods for bioactive compounds from *S. rugosoannulata* and *M. procera*.

### 4.1. Polysaccharide Extraction

Polysaccharides are the most predominant and extensively studied bioactive components in *S. rugosoannulata* and *M. procera*, several methods are employed for polysaccharide extraction, including hot water extraction, solvent extraction, UAE, and ion-exchange chromatography. These extraction methods can efficiently extract polysaccharides from both *S. rugosoannulata* and *M. procera*. Among them, hot water extraction is environmentally friendly, non-toxic, safe, and cost-effective. Solvent extraction is simple to operate, highly selective, and also cost-effective. Ultrasound extraction (UAE) can enhance extraction efficiency while being environmentally friendly and energy-saving. Ion exchange chromatography offers high selectivity and strong operability. While extraction efficiency varies depending on the method, these techniques generally preserve the fundamental structure of polysaccharides.

Georgiev et al. [80] extracted polysaccharides from fresh *S. rugosoannulata* using a combination of freeze-thawing (30 min) and hot water extraction (30 min, 1:100 *w*/*v*). The combined extracts were centrifuged (3000× *g*, 10 min), concentrated to 15% of the original volume using a rotary evaporator at 60 °C, and precipitated overnight with 90% (*v*/*v*) ethanol at 4 °C. The precipitate was redissolved in ultrapure water, deproteinized enzymatically (neutral protease, 37 °C, 2 h), dialyzed (MWCO 3000 Da) for 24 h, and freeze-dried to obtain crude polysaccharides. For compositional analysis, the crude polysaccharides were further purified using DEAE anion-exchange chromatography (45 mm × 260 mm column, elution with distilled water and a NaCl gradient (0–0.5 M, 1 mL/min)) on an AKTA Pure system. Total polysaccharide content was determined using the phenol-sulfuric acid method. Two fractions, SRF-1 and SRF-2, were collected and freeze-dried. SRF-1, identified as the primary lipid-lowering fraction, was further purified using Sephacryl S-200 HR size-exclusion chromatography (26 mm × 1000 mm column, elution with 0.15 M NaCl, 0.4 mL/min). Two target polysaccharides, SRF-3 and SRF-4, were obtained. SRF-3, containing 95.43% total sugars and 0.2% protein, was composed of mannose, glucose, galactose, and methyl galactose in a molar ratio of 8:12:58:12. Its structure was characterized by an α-D-1,6-Galp and α-D-1,6-Me-Galp backbone with branching at the O-2 position of galactose and minor t-β-D-Manp and t-α-L-Fucp side chains. Liu et al. [56] isolated two structurally distinct glucan polysaccharides, SRP-1 and SRP-2, from *S. rugosoannulata* using macroporous adsorption resin and ion-exchange chromatography. Both polysaccharides contained a (1→6)-α-D-glucan backbone but differed in monosaccharide molar ratios and glycosidic linkages. Both exhibited antioxidant activity.

Georgiev et al. [61] extracted polysaccharides from *M. procera* fruiting bodies. The powdered fruiting bodies were defatted with petroleum ether (40–60 °C, 1:10 *w*/*v*, 2 h), filtered, and the residue was extracted with 80% (*v*/*v*) ethanol (1:10 *w*/*v*, 65 °C, 1 h, then overnight at room temperature). The remaining solid was extracted again with 80% ethanol (1:10 *w*/*v*, 2 h, room temperature) and then with 100% acetone (1:10 *w*/*v*, 1 h) before air-drying. Polysaccharides (PSC) were extracted from the alcohol-insoluble solid (AIS) with boiling ultrapure water (1:28 *w*/*v*, two extractions, 1 h each). The extracts were filtered, the residue was washed, and the combined filtrate was centrifuged (4000× *g*, 25 min, room temperature). The supernatant was concentrated, centrifuged again, and precipitated with 95% (*v*/*v*) cold ethanol (1:4 *v*/*v*) at 4 °C overnight. The precipitate was recovered by centrifugation (4000× *g*, 30 min, 4 °C), dissolved in ultrapure water, dialyzed against deionized water (72 h, 4 °C), centrifuged to remove any insoluble material, filtered, and freeze-dried to obtain *M. procera* polysaccharides (MP-PSC). MP-PSC primarily contained neutral polysaccharides rich in Gal (62.3% *w*/*w*) and 3-O-methylated Gal, along with water-soluble α-glucans and/or glycogen. The (hetero) β-glucans contained 1,4,6-β-D-Manp structures.

For the extraction of polysaccharides, different methods yield varying contents of extracted substances. Hot water extraction can significantly enhance the biological activity of *S. rugosoannulata*, achieving a total sugar extraction of 95.43% [80], which is the highest extraction rate, although the crude polysaccharide has a smaller molecular weight. The polysaccharide extracted from *M. procera* (MP-PSC) using solvent extraction contains a high proportion of Gal (62.3% *w*/*w*) [61]. Ion exchange chromatography isolates two antioxidant polysaccharides from *S. rugosoannulata*, with total sugar content ranging from 90.34% to 91.23% [56].

UAE can enhance polysaccharide extraction efficiency. Optimal conditions for *S. rugosoannulata* fruiting bodies (62 °C, 1:30 *w*/*v*, 62 min) yielded 13.25% polysaccharides, while optimal conditions for mycelium (63.1 °C, 1:15 *w*/*v*, 16.33 min) yielded 22.37% [81]. Lu et al. [57] compared hot water extraction and UAE for *S. rugosoannulata* polysaccharides using different particle sizes. Hot water extraction resulted in higher yields and lower molecular weight polysaccharides. Antioxidant activity was not directly correlated with molecular weight. UAE with moderately ground mushroom powder (around 90 μm) enhanced free radical scavenging capacity. Different extraction methods did not disrupt the fundamental structure of *S. rugosoannulata* polysaccharides.

### 4.2. Extraction of Other Bioactive Compounds

Besides polysaccharides, researchers have also investigated the extraction of polyphenols, amino acids, and other bioactive compounds. Common procedures involve solvent extraction of dried mushroom powder, filtration, evaporation, drying, and low-temperature storage. Water and methanol are the primary solvents used. Other techniques, such as MAE and Liquid chromatography-mass spectrometry (LC-MS), are also employed [64,79]. Different solvents, including water, ethanol, and methanol, influence polyphenol extraction efficiency from *M. procera* (<50%, 60–80%, and 60–90%, respectively). MAE significantly enhances polyphenol and amino acid extraction [66,79]. Erbiai et al. [64] identified 35–51 different biomolecules, including sugars, amino acids, fatty acids, and organic acids, in derivatized methanol extracts of *M. procera* using GC, providing insights into its medicinal quality. LC-MS analysis revealed the content and structural characteristics of various phenolic compounds, including cinnamic acid, ferulic acid, and gallic acid [64].

## 5. Omics Analysis

*S. rugosoannulata* and *M. procera*, as important edible fungi, are subjects of increasing “omics” research, providing valuable data on their biological characteristics. These studies not only advance fundamental scientific understanding but also provide a scientific basis for the industrial production of edible fungi. This foundation holds promise for developing superior cultivars and promoting the sustainable development of the edible mushroom industry.

**Figure 5:**
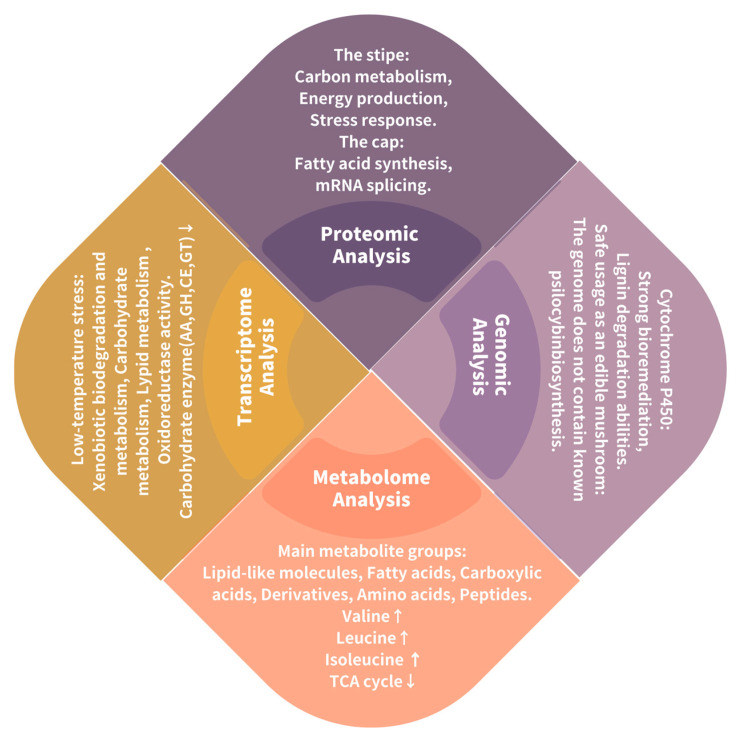
Overview of omics analysis of *S. rugosoannulata.* ↑ indicates promotion, ↓ indicates inhibition.

Several “omics” studies have investigated *S. rugosoannulata* in recent years (Figure 5). Li et al. characterized a monokaryotic *S. rugosoannulata* strain (A15) and, through proteomic analysis, revealed distinct protein expression profiles between the cap and stipe of *S. rugosoannulata* fruiting bodies [82]. Proteins associated with carbon metabolism, energy production, and stress response were more abundant in the stipe, while those related to fatty acid synthesis and mRNA splicing showed higher expression in the cap. Hao et al. performed a transcriptomic analysis of *S. rugosoannulata* under cold stress, identifying 9499 Differentially expressed genes (DEGs) [76]. Gene Ontology (GO) and Kyoto Encyclopedia of Genes and Genomes (KEGG) enrichment analyses indicated that these DEGs were enriched in xenobiotic biodegradation and metabolism, carbohydrate metabolism, lipid metabolism, and oxidoreductase activity. The study concluded that cold stress reduced the expression of carbohydrate-active enzyme genes, such as those encoding auxiliary activities (AAs), glycoside hydrolases (GHs), carbohydrate esterases (CEs), and glycosyltransferases (GTs), leading to decreased cellulase secretion and reduced carbohydrate metabolism and hyphal growth. Another study evaluated the metabolism and activity of *S. rugosoannulata* protein peptidyl material, identifying the primary metabolites as lipid molecules, fatty acids, carboxylic acids and their derivatives, amino acids, and peptides [83]. KEGG pathway enrichment of differentially expressed metabolites highlighted upregulated pathways like valine, leucine, and isoleucine biosynthesis and downregulated pathways such as the citric acid cycle. Biosynthetic pathways like arginine and proline metabolism were enriched with both upregulated and downregulated differentially expressed metabolites. Wu et al. employed ultra-performance liquid chromatography-tandem mass spectrometry (UPLC-MS/MS) and RNA sequencing (RNA-Seq) for quantitative analysis of flavonoids in *S. rugosoannulata* [84]. They identified 53 flavonoid-related metabolites and 6726 DEGs. KEGG analysis identified 59 structural genes encoding enzymes involved in flavonoid biosynthesis, most of which were upregulated during fruiting body development, consistent with flavonoid accumulation. This study established a comprehensive transcriptional and metabolic regulatory network of flavonoids, their biosynthetic enzymes, and transcription factors. A high-quality genome assembly of a Chinese *S. rugosoannulata* cultivar revealed the expansion of genes encoding Manganese peroxidases (MnPs), lignin and xenobiotic degrading enzymes, and cytochrome P450s involved in xenobiotic metabolism, potentially explaining its strong bioremediation and lignin degradation capabilities [85]. The genome lacked genes for known psilocybin biosynthesis, supporting its safe consumption. This genomic analysis is expected to facilitate fruiting body cultivation and provide insights into its bioremediation applications. Finally, Gong et al. investigated the effects of *S. rugosoannulata* cultivation on woodland soil and bacterial communities [9]. Organic matter content was identified as the primary factor influencing soil bacterial community composition, and spent mushroom compost from *S. latifolia* cultivation, rich in organic matter and mycelia, was shown to improve soil nutrient content, bacterial community composition, and diversity.

## 6. Bioactivities and Health-Promoting Benefits

The diverse bioactive compounds in *S. rugosoannulata* and *M. procera* have led to extensive research, revealing a range of properties, including antioxidant, immunomodulatory, anticancer, antibacterial, anti-inflammatory, hypoglycemic, hepatoprotective, and cardiovascular benefits, highlighting their medicinal potential.

### 6.1. Antioxidant Properties

Recent studies demonstrate significant antioxidant activity in both *S. rugosoannulata* and *M. procera*, primarily assessed through free radical scavenging, metal chelation, and lipid peroxidation inhibition assays. *S. rugosoannulata* extracts exhibit notable 1, 1-Diphenyl-2-picrylhydrazyl radical 2, 2-Diphenyl-1-(2, 4, 6-trinitrophenyl) hydrazyl (DPPH) and 2, 2′-Azinobis-(3-ethylbenzthiazoline-6-sulphonate) (ABTS) radical scavenging capacity [3]. Liu et al. [56] reported high DPPH radical scavenging rates (46.67–62.50%) at high extract concentrations, indicating substantial antioxidant potential. *M. procera* extracts also demonstrate strong free radical scavenging activity (IC_50_ = 311.40 μg/mL) [79]. Iron chelation capacity increases with increasing extract concentration for both water and ethanol extracts of *S. rugosoannulata* [46], suggesting that metal chelation contributes to its antioxidant effects. *M. procera* extracts exhibit slightly higher iron chelating activity compared to *S. rugosoannulata*, potentially due to higher concentrations of phenolic and flavonoid compounds [79].

Lipid peroxidation, a key step in cellular oxidation processes, is commonly assessed using the thiobarbituric acid reactive substances (TBARS) assay. *S. rugosoannulata* extracts effectively inhibit β-carotene bleaching, indicating their ability to scavenge free radicals and inhibit lipid peroxidation [46]. *M. procera* also effectively inhibits lipid peroxidation, with its phenolic compounds donating hydrogen atoms to free radicals, thereby disrupting the chain reaction of lipid peroxidation [77,79].

### 6.2. Immunomodulatory Effects

**Figure 6:**
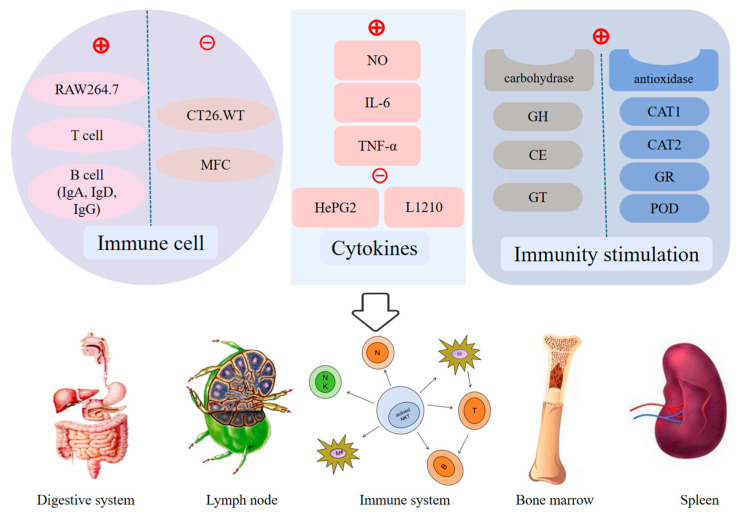
Immunomodulatory mechanism of *S. rugosoannulata* and *M. procera*. *S. rugosoannulata* and *M. procer* promote immune response by enhancing the proliferation of T lymphocytes, B lymphocytes, and RAW264.7 cells while inhibiting the proliferation of immune cells such as CT26.WT and MFC. They also stimulate the production of cytokines like NO, IL-6, and TNF-α, while suppressing the proliferation of substances from HepG2 and L1210 cells. Additionally, they activate immune responses through various immune stimulators, including GH, CE, GT, CAT1, CAT2, GR, and POD. RAW264.7: Mouse macrophage leukemia cells; IgA/D/G: Immunoglobulin A/D/G; CT26.WT: Mouse colon cancer cells; MFC: Mouse gastric cancer cells; NO: Nitric Oxide; IL-6: Interleukin-6; TNF-α: Tumor necrosis factor-alpha; HepG2: Human liver cancer cells; L1210: Mouse leukemia cells; GH: Glycosyl hydrolases; CE: Carbohydrate esterases; GT: Glycosyl transferases; CAT1/2: Catalase1/2; GR: Glutathione reductase; POD: Peroxidase.

*S. rugosoannulata* and *M. procera*, as traditional edible mushrooms, have garnered attention for their potential immunomodulatory effects. Studies suggest that these mushrooms can influence immune cell function, regulate cytokine production, and activate immune-stimulating mechanisms. Immune cells, including T cells, B cells, and macrophages, play central roles in immune responses. *S. rugosoannulata* polysaccharide SR1 stimulates the growth of T lymphocytes, B lymphocytes, and RAW264.7 cells and enhances IgD and IgG secretion by B lymphocytes [3], thereby enhancing cellular immune responses (Figure 6). *M. procera* also enhances immune cell function, with its water extract increasing T cell activity and cytokine secretion, demonstrating immunostimulatory effects. Cytokines are crucial signaling molecules in the immune system, regulating immune cell function and immune responses. *S. rugosoannulata* extracts exhibit positive effects on cytokine regulation, inhibiting the proliferation of HepG2 and L1210 cells potentially enhancing immune responses, and suppressing inflammation [86] (Figure 6). *M. procera* extracts also demonstrate significant cytokine regulatory effects. Water extracts of *M. procera* induce NO, IL-6, and TNF-α production in RAW264.7 cells [61], suggesting potential roles in enhancing immune responses and modulating inflammation. The immunostimulatory mechanisms of *S. rugosoannulata* are primarily attributed to its polysaccharides (Figure 6). Carbohydrate-active enzymes like glycosyl hydrolases (GH), carbohydrate esterases (CE), and glycosyltransferases (GT) contribute to polysaccharide metabolism, while antioxidant enzymes like catalase (CAT1 and CAT2), glutathione reductase (GR), and peroxidase (POD) protect immune cells from oxidative stress [76]. The immunomodulatory effects of *M. procera* are mediated by polysaccharides and other bioactive components. Studies indicate that *M. procera* polysaccharides exert strong immunostimulatory effects by regulating cytokines and activating macrophages [87]. Certain components of *M. procera* can also modulate gut microbiota, promoting the growth of beneficial bacteria and further enhancing host immune function.

### 6.3. Anticancer Properties

**Figure 7:**
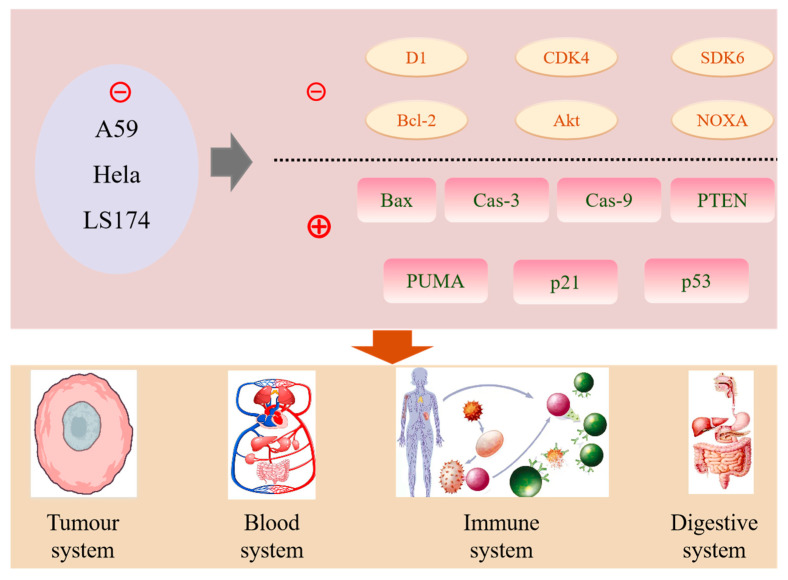
Anticancer mechanism of *S. rugosoannulata* and *M. procera*. *S. rugosoannulata* and *M. procer* exert their anticancer effects by inhibiting the proliferation of A59, HeLa, and LS174 cells. This is primarily reflected in the suppression of the expression of genes such as D1, CDK4, SDK6, Bcl-2, Akt, and NOXA, while promoting the expression of genes including Bax, Caspase-3/9, PTEN, PUMA, and p21/p53. A59: Human non-small cell lung cancer cells; Hela: Human cervical cancer; LS174: Human colon cancer; D1: Cyclin D1; CDK4: Cyclin-dependent kinase 4; Bcl-2: B-cell lymphoma 2; Akt: Protein kinase B; NOXA: Apoptosis regulator; Bax: Bcl-2-associated X protein; Cas-3/9: Caspase-3/9; PTEN: Phosphatase and tensin homolog; PUMA: p53 upregulated modulator of apoptosis; p21/53: Cyclin-dependent kinase inhibitor p21/53.

*S. rugosoannulata* and *M. procera* exhibit promising antitumor activities, including antiproliferative, pro-apoptotic, and antimetastatic effects (Figure 7). Antiproliferative activity refers to the inhibition of cancer cell growth and division. Studies demonstrate that *S. rugosoannulata* extracts exert significant antiproliferative activity against cancer cells. Zhang et al. [86] isolated a novel lectin from *S. rugosoannulata* with antiproliferative activity against cancer cells. Wang et al. [88] further demonstrated the anticancer potential of *S. rugosoannulata*. *M. procera* also exhibits strong antiproliferative activity (Figure 7). Seçme et al. [89] reported that *M. procera* extracts inhibited the proliferation of A549 human lung cancer cells, with an IC_50_ of 2 mg/mL after 72 h. Treatment with *M. procera* extracts downregulated the expression of cyclin D1, CDK4, CDK6, Bcl-2, Akt, and NOXA genes and upregulated the expression of Bax, caspase-3, caspase-9, PTEN, PUMA, p21, and p53 genes. Kosanić et al. [79] reported cytotoxic activity against HeLa (human cervical cancer), A549, and LS174 (human colon cancer) cells, with IC50 values ranging from 19.01 to 80.27 μg/mL. Zara et al. [90] identified *M. procera* extracts as potential inhibitors of cancer cell proliferation (IC_50_ = 6.18 μg/mL) and, through molecular docking studies targeting glucose-6-phosphate dehydrogenase (G6PD), identified *p*-hydroxybenzoate, quercetin, and syringic acid as compounds with strong binding affinity to G6PD.

The pro-apoptotic activity involves inducing programmed cell death in cancer cells. *S. rugosoannulata* extracts can promote apoptosis by inducing cell cycle arrest, activating apoptotic pathways, and triggering oxidative stress. These extracts may generate free radicals and induce oxidative stress, leading to cellular damage and activation of apoptotic signaling pathways [62]. *M. procera* also exhibits pro-apoptotic activity, with its extracts modulating apoptotic signaling pathways. Seçme et al. [89] reported a 28% increase in apoptosis in *M. procera* extract-treated cells using the TUNEL assay.

Antimetastatic activity refers to the inhibition of cancer cell metastasis. While research on the antimetastatic effects of *S. rugosoannulata* is limited, *M. procera* has shown promising results. Kosanić et al. [79] found that *M. procera* extracts significantly inhibited the migration of LS174 human colon cancer cells. Seçme et al. [89] reported that *M. procera* extracts effectively reduced the invasiveness of A549 lung cancer cells.

### 6.4. Antimicrobial Activity

*S. rugosoannulata* and *M. procera* possess significant antibacterial and antifungal properties, offering potential applications in food safety and medicine. Aqueous extracts of *S. rugosoannulata* effectively inhibit the growth of *Escherichia coli*, *Staphylococcus aureus*, and *Bacillus subtilis*. The polysaccharides and phenolic compounds in these extracts significantly reduce bacterial growth rates. Wu et al. [16] isolated a unique sterol from *S. rugosoannulata* with neuroprotective and anti-staphylococcal activity. *S. rugosoannulata* also exhibits antifungal activity, with extracts effectively inhibiting the growth of yeast and other fungi [91]. These antimicrobial and antifungal effects are attributed to the action of bioactive compounds and immunomodulatory mechanisms. The polysaccharides, phenols, and flavonoids in *S. rugosoannulata* exert antimicrobial activity by damaging cell membranes and inhibiting microbial metabolism. *S. rugosoannulata* extracts can also enhance immune responses, increasing macrophage phagocytic activity, which may play a role in systemic infections.

*M. procera* also demonstrates strong antimicrobial activity. Studies indicate that its extracts inhibit various pathogenic bacteria [79]. Bioactive components like polyphenols and coumarins are considered key contributors to its antimicrobial activity. *M. procera* also exhibits significant antifungal activity, effectively inhibiting the growth and biofilm formation of various pathogenic fungi [92]. Its antimicrobial and antifungal mechanisms involve the action of bioactive components and biofilm inhibition. Polyphenols and antioxidants in *M. procera* disrupt microbial cell walls and membranes, inhibiting growth. These components can also bind to proteins, affecting microbial physiological functions. Furthermore, *M. procera* inhibits fungal biofilm formation.

### 6.5. Others

The anti-inflammatory properties of *S. rugosoannulata* are primarily attributed to its rich array of bioactive compounds. Studies have shown that triterpenoids in *S. rugosoannulata* exhibit potent anti-inflammatory activity [69]. *M. procera* also demonstrates anti-inflammatory effects. Bioactive components like flavonoids and polyphenols exert antioxidant effects, reducing oxidative stress and mitigating inflammation-related damage [93].

Research on the hypoglycemic effects of *S. rugosoannulata* is ongoing, but preliminary findings are promising. Zhai et al. [94] reported that *S. rugosoannulata* exopolysaccharides have the potential to prevent hyperglycemia in diabetic rats. *M. procera* exhibits more established hypoglycemic effects. Studies have shown that *M. procera* extracts can effectively inhibit key enzymes involved in diabetes [77]. Furthermore, *M. procera* may enhance hepatic glucose utilization, contributing to its hypoglycemic effects.

The hepatoprotective effects of *S. rugosoannulata* involve preventing liver damage and promoting liver cell regeneration. Studies indicate that *S. rugosoannulata* extracts can reduce liver enzyme levels, suggesting efficacy in mitigating liver injury. This effect may be linked to its antioxidant components, which scavenge free radicals and reduce oxidative stress-induced damage to liver cells [80]. Polysaccharide extracts from *S. rugosoannulata* significantly increased HepG2 cell viability and reduced extracellular alanine aminotransferase (ALT) and intracellular triglyceride (TG) levels, suggesting that SRF-1 can protect against free fatty acid (FFA)-induced liver cell damage and lipid accumulation [80]. *M. procera* also exhibits hepatoprotective properties, with extracts reducing hepatic fat deposition and improving liver function. Its polysaccharides have been shown to decrease lipid peroxidation levels in liver tissue, reducing the risk of oxidative damage [61]. *M. procera* may also regulate the expression of lipid metabolism-related enzymes in the liver, supporting liver cell health.

*S. rugosoannulata* is believed to have cardioprotective effects. Studies have shown that its extracts can improve blood lipid profiles, lowering low-density lipoprotein (LDL) cholesterol and increasing high-density lipoprotein (HDL) cholesterol, thereby reducing the risk of cardiovascular disease [69]. Furthermore, polyphenols in *S. rugosoannulata* may improve vascular endothelial function, contributing to better blood pressure regulation. *M. procera* also exhibits cardioprotective potential. Studies suggest that it can lower blood pressure and improve blood lipid profiles [65]. Its antioxidant components can reduce oxidative stress, preventing atherosclerosis. These combined mechanisms support the potential of *M. procera* as a cardioprotective food.

## 7. Safety and Toxicology

As two commonly consumed edible mushrooms, the safety of *S. rugosoannulata* and *M. procera* is of significant interest. This section discusses their safety and potential toxicity, considering factors such as heavy metal accumulation, anti-nutritional factors, improper storage, and processing and cooking methods.

### 7.1. Heavy Metal Accumulation

*S. rugosoannulata*, which prefers acidic soils and forms symbiotic relationships with conifers like pine trees, generally accumulates lower levels of heavy metals. Studies indicate that lead and cadmium levels in *S. rugosoannulata* are typically below safety limits, making it a safe dietary choice. *M. procera*, which grows in grasslands and drier soils, generally exhibits a greater capacity for heavy metal accumulation. Under certain conditions, it can accumulate potentially harmful levels of lead, cadmium, and mercury [95,96]. Wild *M. procera* can effectively absorb certain metals (Cd, Cu, K, Mg, Na, and Zn) naturally present in the mycelial substrate. While the fruiting bodies of *M. procera* provide various nutrients after cooking, they can also contain toxic metals like cadmium, lead, and mercury. Considering the observed levels of these toxic metals and their recommended intake limits, consumption of *M. procera* should be limited to one or two meals per week [96]. *M. procera* accumulates significant amounts of Cd and Cr, with Cd accumulation in the cap being up to five times higher than in the stipe. Frequent consumption can negatively impact human health, with children being 1.5 times more susceptible than adults [97].

### 7.2. Antinutritional Factors

While *S. rugosoannulata* and *M. procera* are nutritious edible mushrooms, they may contain antinutritional factors that can interfere with nutrient absorption. *S. rugosoannulata* contains a lectin (SRL), a 38 kDa protein with a unique N-terminal sequence, which exhibits activity against HepG2 liver cancer cells, L1210 leukemia cells, and HIV-1 reverse transcriptase [86]. While this lectin may offer health benefits, *S. rugosoannulata* also contains oxalates, which can bind to calcium, forming insoluble calcium oxalate and potentially leading to mineral deficiencies. Excessive intake of fiber and insoluble polysaccharides can also hinder nutrient absorption and cause digestive discomfort. Furthermore, *S. rugosoannulata* may contain anti-enzymes, such as amylase and protease inhibitors, which can interfere with nutrient digestion and absorption.

Lectins with potential health-promoting effects, including polysaccharide–protein and polysaccharide–peptide complexes, have been identified in *M. procera* fruiting bodies. These lectins have garnered attention for their anticancer, immunomodulatory, and antiviral properties, although further research is needed to fully elucidate their structure and function [98]. *M. procera* may also contain oxalates, which can affect calcium bioavailability. The presence of compounds that inhibit digestive enzymes may reduce nutrient absorption efficiency. Polyphenols, while possessing antioxidant activity, can potentially inhibit the absorption of minerals like iron and zinc.

### 7.3. Improper Storage

Improper storage of *S. rugosoannulata* and *M. procera* can lead to contamination by bacteria like *E. coli* and molds like *Aspergillus flavus* and *Aspergillus niger*. Bacterial growth leads to spoilage, while mold growth can produce mycotoxins, posing health risks. Spoilage also degrades flavor and texture, leading to unpleasant sensory experiences and potential gastrointestinal issues like nausea, vomiting, and abdominal pain. Furthermore, improper storage can exacerbate the risk of allergic reactions in sensitive individuals.

Fresh mushrooms can maintain their freshness to the greatest extent when stored under refrigeration at 0–4 °C. The humidity should be kept at around 85–90% to prevent drying out. It is essential to ensure good ventilation during storage to avoid water accumulation and mold growth. Do not wash the mushrooms before storage to reduce moisture and prevent spoilage. It is best to use paper bags for storage to avoid crushing. Aim to consume them while they are still in high freshness.

### 7.4. Mitigation Strategies

Consuming *S. rugosoannulata* and *M. procera* requires caution. Risks can be mitigated by proper identification, sourcing from reputable suppliers, understanding potential toxicity, and careful processing and cooking. Familiarization with mushroom characteristics (color, shape, odor, growing environment) and distinguishing edible species from toxic look-alikes is crucial. For wild mushrooms, consultation with expert foragers is essential. Awareness of potential toxicities, allergies, and sensitivities is also important. Appropriate processing can reduce the risks associated with heavy metals and anti-nutritional factors. Improperly stored mushrooms should be discarded. Optimal processing and cooking methods maximize the retention of beneficial compounds.

## 8. Conclusions and Prospects

This review has provided a comprehensive overview of the current knowledge regarding the nutritional composition, bioactive compounds, extraction methods, bioactivities, and safety considerations of *S. rugosoannulata* and *M. procera*. Both species demonstrate considerable nutritional value, offering a rich source of proteins, essential amino acids, dietary fiber, vitamins, and minerals. Moreover, they harbor a diverse array of bioactive compounds, including polysaccharides, phenolic compounds, terpenes, peptides, and enzymes, which contribute to their remarkable range of health-promoting properties. These include antioxidant, immunomodulatory, anticancer, antimicrobial, anti-inflammatory, hypoglycemic, hepatoprotective, and cardiovascular benefits. While both species offer significant promise as functional foods and sources of natural medicines, careful consideration of potential heavy metal accumulation, anti-nutritional factors, and proper storage practices is essential to ensure consumer safety.

While this review highlights the promising nutritional and therapeutic potential of *S. rugosoannulata* and *M. procera*, several key areas require further investigation to fully capitalize on their beneficial properties. A deeper exploration of the structural characterization of bioactive compounds, particularly polysaccharides, is crucial. This includes determining precise monosaccharide compositions, glycosidic linkages, and branching patterns, which can significantly influence their biological activity. Further research should also focus on optimizing extraction methods for specific bioactive components. While several methods have been employed, including solvent extraction, hot water extraction, and ultrasound-assisted extraction, there is a need for standardized and efficient protocols that maximize yields while preserving the integrity and bioactivity of the target compounds. Furthermore, a more comprehensive understanding of the structure–activity relationships of these bioactive compounds is essential. This involves investigating how specific structural features of polysaccharides, phenolic compounds, terpenes, and peptides contribute to their observed antioxidant, immunomodulatory, anticancer, and antimicrobial activities. Such knowledge is crucial for developing targeted and effective therapeutic interventions. While in vitro and some in vivo studies have demonstrated promising bioactivities, there is a critical need for well-designed clinical trials to validate these findings in humans. This will provide robust scientific evidence for the efficacy of *Stropharia*- and *Macrolepiota*-derived compounds in preventing and treating various diseases. Finally, investigating the potential synergistic effects of different bioactive compounds within these mushrooms could lead to the development of more potent and effective formulations for therapeutic applications. This includes exploring combinations of polysaccharides, phenolic compounds, and other bioactive components to maximize their combined health benefits. By addressing these research priorities, we can unlock the full therapeutic potential of *S. rugosoannulata* and *M. procera* and pave the way for their integration into evidence-based health-promoting strategies.

## Figures and Tables

**Table 1:** Nutritional composition of *S. rugosoannulata* and *M. procera*.

Nutritional Components	*S. rugosoannulata*	*M. procera*	Refs
Moisture	90–93%	88–92%	[3]
Proteins	17.99–25.67%	7.62–39.68%	[26,27]
Carbohydrates	45.17–54.60%	40.90–60.30%	[28,29,30]
Lipids	1.33–2.30% (over 77% unsaturated fatty acids, including 57% linoleic acid and 13% palmitic acid)	0.70–4.23% (rich in unsaturated fatty acids, predominantly linoleic acid)	[29,31]
Macroelements	K (1600 mg/100 g), P (75–100 mg/100 g), Mg (20–30 mg/100 g), Ca (70–80 mg/100 g)	K (300–500 mg/100 g), Mg (15–25 mg/100 g), Ca (400–500 mg/100 g)	[32,33,34]
Trace elements	Fe (19.5–24.5 mg/100 g), Zn (5.5–10.0 mg/100 g), Se (0.1–0.5 mg/100 g)	Fe (1.5–3 mg/100 g) Zn (0.5–1.2 mg/100 g)	[35,36,37,38,39]
Vitamins	B vitamins (B2, B3, B5, B6, B9, and B12), Vitamin C, E, K, Provitamin D2, Beta-carotene	B vitamins (B2, B3, and B5), Vitamin D, E, K, Beta-carotene	[20,40,41,42,43]
Essential amino acids	6.43–11.70%, with isoleucine being the most abundant.	Relatively low	[26,44,45,46]
Nonessential amino acids	2.88–6.84% glutamic acid and 1.72–3.07% aspartic acid	Alanine (1.10 g/100 g)	[27,45,47]

**Table 2:** Extraction and isolation methods for functional bioactive compounds in *S. rugosoannulata* and *M. procera*.

Method	Characteristics	Extracted Compounds	Refs
Solvent extraction	Simple operation and high selectivity, but solvent residue and long extraction time are limitations. Optimization of solvent polarity and concentration can improve performance.	Polysaccharides, Gal (62.3% *w*/*w*); phenolics, 60–90%, protocatechuic acid 2.23–2.25 µg/g dw, cinnamic acid 8.67 µg/g dw	[79]
Hot water extraction	Eco-friendly and non-toxic, but with low efficiency and prolonged duration. Extraction temperature and time can be optimized to enhance outcomes.	Polysaccharides, 95.43% total sugar, mannose:glucose:galactose:methylgalactose = 8:12:58:12	[57,80]
Ultrasound-assisted extraction (UAE)	Increasing extraction efficiency and purity but requiring specialized equipment and showing energy-intensive. Frequency and extraction time can be optimized for better results.	Polysaccharides, 13.25–22.37%	[81]
Microwave- assisted extraction (MAE)	Fast and efficient, suitable for thermosensitive compounds. However, uneven heating may impact the extraction process. Extraction power and time can be optimized to mitigate this issue.	Polyphenols, 1.22–6.80%; amino acids, 3.57–23.06%, alanine 0.41%, threonine 1.26%, tryptophan 2.82%	[64,79]
Ion exchange chromatography	High selectivity and effective for biomacromolecule separation with strong operability. However, it demands strict sample conditions, is unsuitable for non-ionic compounds, and has a slow separation rate.	Polysaccharides, 90.34–91.23% total sugar, (1→, 6)-α-D-dextran cytoskeleton	[56]
